# The mtDNA nt7778 G/T Polymorphism Augments Formation of Lymphocytic Foci but Does Not Aggravate Cerulein-Induced Acute Pancreatitis in Mice

**DOI:** 10.1371/journal.pone.0102266

**Published:** 2014-07-10

**Authors:** Sarah Müller, Burkhard Krüger, Falko Lange, Cristin N. Bock, Horst Nizze, Änne Glass, Saleh M. Ibrahim, Robert Jaster

**Affiliations:** 1 Department of Medicine II, Division of Gastroenterology, University Medicine Rostock, Rostock, Germany; 2 Division of Medical Biology, University Medicine Rostock, Rostock, Germany; 3 Oscar-Langendorff-Institute of Physiology, University Medicine Rostock, Rostock, Germany; 4 Institute of Pathology, University Medicine Rostock, Rostock, Germany; 5 Institute for Biostatistics and Informatics in Medicine and Ageing Research, University Medicine Rostock, Rostock, Germany; 6 Department of Dermatology, University of Lübeck, Lübeck, Germany; University of Szeged, Hungary

## Abstract

A polymorphism in the *ATP synthase 8* (*ATP8*) gene of the murine mitochondrial genome, G-to-T transversion at position 7778, has been suggested to increase susceptibility to multiple autoimmune diseases, including autoimmune pancreatitis (AIP). The polymorphism also induces mitochondrial reactive oxygen species generation, secretory dysfunction and β-cell mass adaptation. Here, we have used two conplastic mouse strains, C57BL/6N-mtAKR/J (B6-mtAKR; nt7778 G; control) and C57BL/6N-mtFVB/N (B6-mtFVB; nt7778 T), to address the question if the polymorphism also affects the course of cerulein-induced acute pancreatitis in mice. Therefore, two age groups of mice (3 and 12-month-old, respectively) were subjected to up to 7 injections of the secretagogue cerulein (50 µg/kg body weight) at hourly intervals. Disease severity was assessed at time points from 3 hours to 7 days based on pancreatic histopathology, serum levels of α-amylase and activities of myeloperoxidase (MPO) in lung tissue. A comparison of cerulein-induced pancreatic tissue damage and increases of α-amylase and MPO activities showed no differences between the age-matched groups of both strains. Interestingly, histological evaluation of pancreatic tissue of both untreated and cerulein-treated B6-mtAKR and B6-mtFVB mice also revealed the presence of infiltrates of immune cells surrounding ducts and vessels; a finding that is compatible with an early stage of AIP. After recovery from cerulein-induced pancreatitis (day 7 after the injections), 12-month-old B6-mtFVB mice but not B6-mtAKR mice displayed aggravated lymphocytic lesions. A comparison of 12-month-old mice with other age groups of both strains revealed that lymphocytic foci were largely absent in 3-month-old mice, while 24-month-old mice were more affected. Together, our data suggest that the mtDNA nt7778 G/T polymorphism does not aggravate cerulein-induced acute pancreatitis. Autoimmune-like lesions, however, may progress faster if additional tissue damage occurs.

## Introduction

A growing body of evidence suggests that mitochondrial dysfunctions are crucially involved in the development of acute pancreatitis (AP) [1], a potentially deadly disease that is among the leading causes of hospitalization for gastrointestinal disorders worldwide [2], [3]. Thus, recent studies have shown that the most common factors that induce pancreatitis cause mitochondrial damage with a consequent impairment of energy metabolism and depletion of ATP [4]–[10]. Mitochondria are also one of the major sources of reactive oxygen species (ROS), which have been implicated in the pathogenesis of AP in numerous studies [11]–[14]. In the context of AP, mitochondrial injury can induce apoptosis via cytochrome c release and activation of the caspase cascade, or necrosis via the opening of mitochondrial permeability transition pore, thereby contributing to cell death and the development of pancreatic tissue damage [1].

Mitochondria harbor a closed, circular, double-stranded DNA termed the mitochondrial genome that encodes, for example, for parts of the mitochondrial oxidative phosphorylation enzyme complexes [15]. In humans and animals, mitochondrial DNA (mtDNA) variations have been identified that cause maternally inherited mtDNA disorders or lead to functional changes which predispose individuals for severe diseases [16], [17]. A systematic analysis of mtDNA variations, however, is hampered by the fact that targeted mutation of the mitochondrial genome currently remains technically difficult due to lack of recombination and the possibility of heteroplasmy. This is one of the reasons why data on the role of mitochondrial DNA polymorphisms in the pathogenesis of pancreatitis are still very rare.

Here, we have compared the susceptibility of two conplastic mouse strains, C57BL/6N-mtFVB/N (B6-mtFVB) and C57BL/6N-mtAKR/J (B6-mtAKR), to develop pancreatitis. The strains have previously been established by crossing mitochondrial genomes of common inbred strains on the C57BL/6N background and differ in just one position of their mitochondrial genome, nt7778: While B6-mtAKR mice carry the wild-type sequence (nt7778 G), the strain B6-mtFVB harbors the naturally occurring nt7778T polymorphism inside the mitochondrial encoded *Atp8* gene that causes an amino acid exchange from Asp to Tyr [18]–[20]. ATP8 represents a subunit of the ATP synthase (complex V) and has been implicated in the correct assembly of ATP synthase holoenzyme [21].

In previous studies, a variety of pathophysiological consequences of the mtDNA nt7778 G/T polymorphism has been described. Thus, in β-cells of the endocrine pancreas, mitochondrial ROS generation, secretory dysfunction and cell mass adaptation were observed [20]. Interestingly, in a murine model of acute endotoxemic liver failure, the polymorphism was shown to *improve* the hepatic energy status [22]. The nt7778 G/T also affects anxiety-like behavior as well as the hormonal response to psychological stress [23]. A particular motivation for this study came from a recent report that showed an increased susceptibility of B6-mtFVB mice to multiple autoimmune diseases, including autoimmune pancreatitis (AIP) [19]. AIP represents a rare form of chronic pancreatitis (CP) that has drawn a lot of attention in recent years due to the fact that (1) AIP represents a differential diagnosis of pancreatic cancer and (2) AIP, in contrast to other forms of CP, responds to steroid treatment [24]. To the best of our knowledge, it has not been studied so far if an autoimmune-prone background affects the course of AP and vice versa. To address this question, we have employed the model of cerulein-induced AP in mice. The results show that the mtDNA polymorphism nt7778 G/T, neither in 3 nor in 12-month-old mice, has an effect on the severity of cerulein-induced AP. The data, however, also indicate that aged B6-mtAKR and B6-mtFVB mice display autoimmune-like pancreatic lesions, but only B6-mtFVB mice develop enlarged lymphocytic foci when challenged with cerulein.

## Materials and Methods

### Reagents

Unless stated otherwise, all reagents were obtained from Sigma-Aldrich (Deisenhofen, Germany).

### Animal Studies

The generation of the conplastic mouse strains used in this study has previously been described [18]–[20]. Briefly, the strains B6-mtFVB and B6-mtAKR were established by crossing females from the mitochondrial donor strain (AKR and FVB) to males with the preferred genomic background (C57BL/6NTac). The female offspring were subsequently backcrossed to males of the recipient strain. After 10 generations, the offspring were regarded as pure conplastic strain [18]–[20].

The animal experiments were approved by the local animal use and care committee (Landesamt für Landwirtschaft, Lebensmittelsicherheit und Fischerei Mecklenburg-Vorpommern, permit number for the study: LALLF M-V/TSD/7221.3-1.1-077/11). The mice had access to water and standard laboratory chow ad libitum. All animals received humane care according to the German legislation on protection of animals and the Guide for the Care and Use of Laboratory Animals (NIH publication 86–23, revised 1985), and all efforts were made to minimize suffering. The cerulein studies were performed with mice of both sexes at an age of 3 or 12 months as indicated. The mice were fasted overnight with free access to water, before the secretagogue cerulein (Bachem, Heidelberg, Germany) was administered in up to seven intraperitoneal injections of 50 µg/kg body weight at hourly intervals [25], [26]. Control mice were also fasted overnight but did not receive cerulein injections. The experimental groups (same strain, age and time of cerulein treatment) usually consisted of 6 mice and in a few cases of 5 individuals. At intervals between 3 hours and 7 days after the first intraperitoneal injection of cerulein, the mice were euthanized by an overdose of ketamine/xylazine mixture followed by cervical dislocation. The pancreas and serum (obtained from whole blood) were harvested and stored under appropriate conditions (−80°C for shock-frozen native tissue) until they were assayed. In some investigations, pancreatic tissue from 24-month-old mice that did not undergo cerulein injections was included.

### Amylase Measurement

Activity of α-amylase in serum was determined in a routine laboratory using the IFCC reference method [27] (AMY reagent; Beckman Coulter, Mervue, Galway, Ireland).

### Histology, Immunohistochemistry and Detection of Apoptotic Cells *in situ*

For histology, pancreata were fixed in 4% formaldehyde phosphate buffer overnight and processed for paraffin embedding. Routine hematoxylin and eosin (H&E) staining for determination of organ inflammation was performed on 1-µm sections using standard procedures.

Histopathological evaluation of cerulein-induced pancreatic injury was conducted by light microscopy as previously described [28]. Therefore, the severity of organ inflammation was determined on blinded samples, employing a scoring system to quantify (1) the degree of edema, (2) the presence of potentially reversible cellular damage, (3) the frequency of dead (apoptotic or necrotic) cells, and (4) the number of infiltrating inflammatory cells. As signs of necrotic cell death, visible ruptures of the cell membrane, cellular disintegration and progressive cell lysis were considered. Apoptotic cells were identified by their pyknotic nuclei, cell fragmentation and the presence of apoptotic bodies. Cells showing swelling, vacuolization and cystic degeneration, but none of the described signs of cell death, were assessed as being damaged in a potentially reversible manner.

Each parameter was evaluated on a scale from 0–3 (0 being normal and 3 being severe; with the possibility of intermediate scores of 0.5, 1.5 and 2.5, respectively), resulting in a maximum total score of 12.

Autoimmune-like pancreatic lesions were evaluated applying a semi-quantitative scoring system to blinded samples as previously described for the scoring of AIP [29], [30]. Briefly, lymphocytic infiltrates were assessed as follows: 0, no pathological changes; 1, presence of mononuclear cells in the interstitium without parenchymal destruction; 2, focal parenchymal destruction with mononuclear cell infiltration; 3, diffuse parenchymal destruction, with residues of intact parenchyma; 4, extended mononuclear cell infiltrates, destruction of acini and (partial) replacement by adipose tissue.

Infiltrating inflammatory cells were classified by immunohistochemical analysis of air-dried cryostat sections (6 µm), applying specific antibodies (anti-CD11b, ImmunoTools, Friesoythe, Germany and anti-CD3, BD Pharmingen, Heidelberg, Germany, respectively) and an avidin–biotin-peroxidase (ABC) technique precisely as described before [28]. Subsequently, the slides were counterstained with Mayer's hemalum solution, dehydrated by four short incubations in ethanol and xylene (two times each) and embedded in Pertex (MEDITE, Burgdorf, Germany). Apoptotic cells in pancreatic tissue were detected employing cryostat sections (6 µm) and the *ApopTag Peroxidase Apoptosis Detection Kit* (Millipore, Billerica, MA, USA). The kit is based on the TUNEL method and stains apoptotic cells *in situ* by labeling DNA strand breaks. Staining was performed according to the instructions of the manufacturer as previously described [28]. Afterwards, tissue sections were counterstained with methyl green at 0.5% (10 min), dehydrated with xylene and embedded in Pertex. Positive-stained cells (ABC staining and ApopTag Kit, respectively) were counted in ten representative areas per section (one area  = 0.09 mm^2^).

### Measurement of Myeloperoxidase (MPO) Activity

MPO activity in lung tissue was determined using the MPO fluorometric detection kit from Enzo Life Sciences (Lörrach, Germany). The kit uses a non-fluorescent detection reagent, which is oxidized in the presence of hydrogen peroxide and MPO to produce its fluorescent analog. The lung tissue was processed by homogenization, solubilization and sonication as previously described [28] and cleared samples were stored at −80°C until assayed. MPO activity was measured in 96-well plates, using 50 µL of sample and 50 µL of *reaction cocktail*. After 60 min of incubation in the dark, fluorescence was recorded employing an *Infinite 200 microplate reader* (Tecan Group Ltd., Männedorf, Switzerland) and an excitation/emission wavelength of 550/595 nm, respectively. MPO activity was calculated using a MPO standard delivered with the kit.

### *In vitro* Studies with Pancreatic Acini

Acini were freshly prepared from the mouse pancreas by digestion with collagenase and suspended in HEPES (24.5 mmol/L)-buffered medium (pH 7.5) containing NaCl (96 mmol/L), KCl (6 mmol/L), MgCl_2_ (1 mmol/L), NaH_2_PO_4_ (2.5 mmol/L), CaCl_2_ (0.5 mmol/L), glucose (11.5 mmol/L), Na-pyruvate (5 mmol/L), Na-glutamate (5 mmol/L), Na-fumarate (5 mmol/L), minimum essential medium (1% v/v), and bovine serum albumin, fraction V (1% w/v) as described before [28].

Prior to the detection of protease activities, acini (biovolume concentration: 2 mm^3^/mL living cells) were treated with cerulein (Bachem) at the supramaximal concentration of 10 nmol/L for up to 90 min at 37°C. Next, acini were washed, resuspended in medium without secretagogue and incubated with the synthetic substrates for trypsin (CBZ-Ile-Pro-Arg)_2_-rhodamine-110 (10 µmol/L) and elastase (CBZ-Ala_4_)_2_-rhodamine-110 (10 µmol/L) (Fisher Scientific, Schwerte, Germany), respectively. Subsequently, cerulein-treated acini and control cultures, together with the substrate, were transferred to 96-well microtiter plates. Substrate cleavage in cerulein-treated acini and control cultures was quantified by cytofluorometry at 485 nm excitation and 530 nm emission wavelengths (CytoFluor 2350; Millipore, Bedford, MA). Therefore, changes of fluorescence intensity (ΔF) at RT were recorded over a time period of 90 min. The data were expressed as ΔF/Δt ratio as previously reported [28], [31].

For the detection of intracellular ROS levels, the cell-permeable non-fluorescent probe 2′,7′-dichlorofluorescin diacetate (DCFH-DA) was employed. In the presence of hydroxyl, peroxyl and other ROS activity, intracellular DCFH-DA, by oxidation and de-esterification, turns to highly fluorescent 2′,7′-dichlorofluorescein. Measurements were performed in black 96-well plates as described before [28]. Therefore, acini were preincubated with DCFH-DA (dissolved in dimethyl sulfoxide) at a final concentration of 50 µmol/L for 30 min at RT. After centrifugation, the cells were resuspended in DCFH-DA-free assay medium (HEPES 24.5 mmol/L; pH 7.4, NaCl 96 mmol/L, KCl 6 mmol/L, MgCl_2_ 1 mmol/L, NaH_2_PO_4_ 2.5 mmol/L, glucose 11.5 mmol/L, CaCl_2_ 0.5 mmol/L, Na-pyruvate 5 mmol/L, Na-glutamate 5 mmol/L, Na-fumarate 5 mmol/L, Eagle medium 10% v/v, bovine serum albumin fraction V 1% w/v) and exposed to cerulein at either supramaximal (10 nmol/L) or maximal (0.1 nmol/L) concentrations for 30 min. Subsequently, fluorescence intensity was determined at 495 nm excitation and 529 nm emission wavelengths (GloMax-Multi+ Detection System, Promega, Madison, WI, USA). Data are expressed as percentage of controls as described in the figure legend.

### Statistical Analysis

All data were stored and analyzed using the IBM SPSS Statistics 22.0. Results are expressed as mean ± standard error of mean (SEM) for the indicated number of animals/samples per experimental protocol. Statistical significance was checked using ANOVA for factors “mouse strain”, “time of cerulein treatment” and “age of mice”. Either a General Linear Model (GLM) procedure (Univariate or Repeated Measurements, depending on experimental design) was applied, or, if assumptions were failed, the respective non-parametric procedure for analysis of variance (Kruskal-Wallis-H or Friedman test). In addition, in GLM there were all effects between groups post-hoc tested by the two-sided Dunnett-T test, and the effects within groups by a simple contrast, with the first category as reference. Otherwise, the Mann-Whitney U-test (Bonferroni-adjusted) was used for post-testing. All subgroups were subsequently tested non-parametrically (Mann-Whitney U-test, Bonferroni-adjusted in case of multiple testing). P<0.05 was considered to be statistically significant.

## Results

### Pancreatic Histopathology of B6-mtAKR and B6-mtFVB Mice

To study the effects of the mtDNA nt7778 G/T polymorphism on the course of AP, B6-mtAKR and B6-mtFVB mice were treated by up to seven intraperitoneal injections of cerulein at hourly intervals.

**Figure 1:**
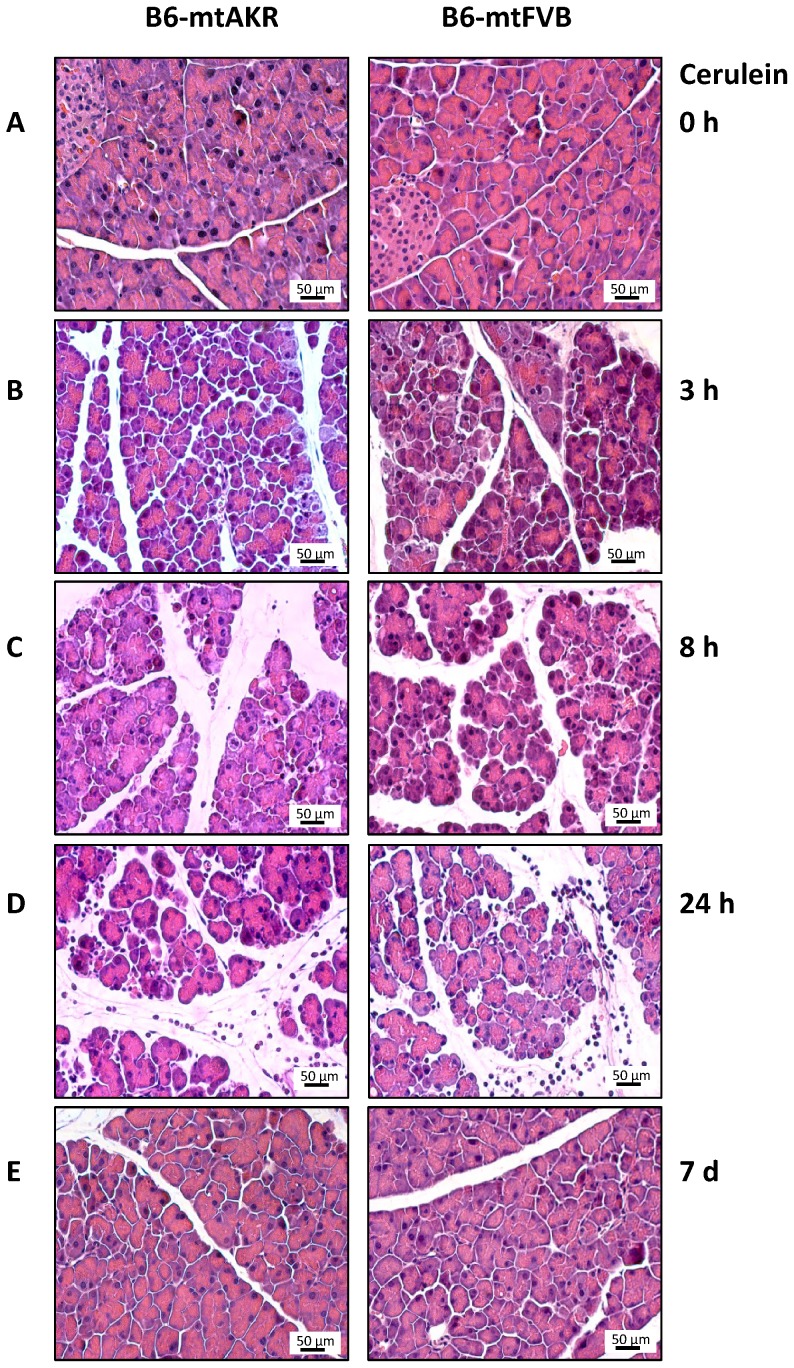
Pancreatic histopathology of cerulein-treated and untreated 12-month-old B6-mtAKR and B6-mtFVB mice. Pancreatic sections were stained with H&E. Photographs A–E display representative examples of pancreatic damage at the time points 0 h, 3 h, 8 h, 24 h and 7 days after the start of cerulein treatment. Left panel: B6-mtAKR mice, right panel: B6-mtFVB mice. The following description of pancreatic histopathology refers to both strains, since major differences were not observed: (A) 0 h – healthy pancreatic tissue (B) 3 h and (C) 8 h – progressive interstitial edema, accompanied by cell swelling, vacuolization and cystic degeneration; death of single cells (D) 24 h – interstitial edema, cell degeneration and multiple apoptotic and necrotic cells; presence of scattered interstitial inflammatory cells (E) day 7 – largely healthy pancreatic tissue. All photographs show selected regions without lymphocytic foci.

The typical pancreatic histopathology of B6-mtAKR and B6-mtFVB mice is shown in Fig. 1 for the example of 12-month-old animals. Untreated mice of both strains presented with largely normal pancreatic tissue (A; for the exception, see section “Lymphocytic foci in B6-mtAKR and B6-mtFVB mice”). At 3, 8 and 24 h after the first injection of cerulein, pancreatic tissue from B6-mtAKR mice (left panels) and B6-mtFVB mice (right panels) showed the characteristic histopathological changes of cerulein-induced AP (B, C and D, respectively). The findings include (1) interstitial edema, (2) cell swelling, vacuolization and cystic degeneration, (3) cell death by apoptosis and necrosis and (4) tissue infiltration with scattered inflammatory cells and reached a maximum after 8–24 h. On day 7, an almost complete regeneration was observed (E). Obvious differences between the two strains were not detected.

**Figure 2:**
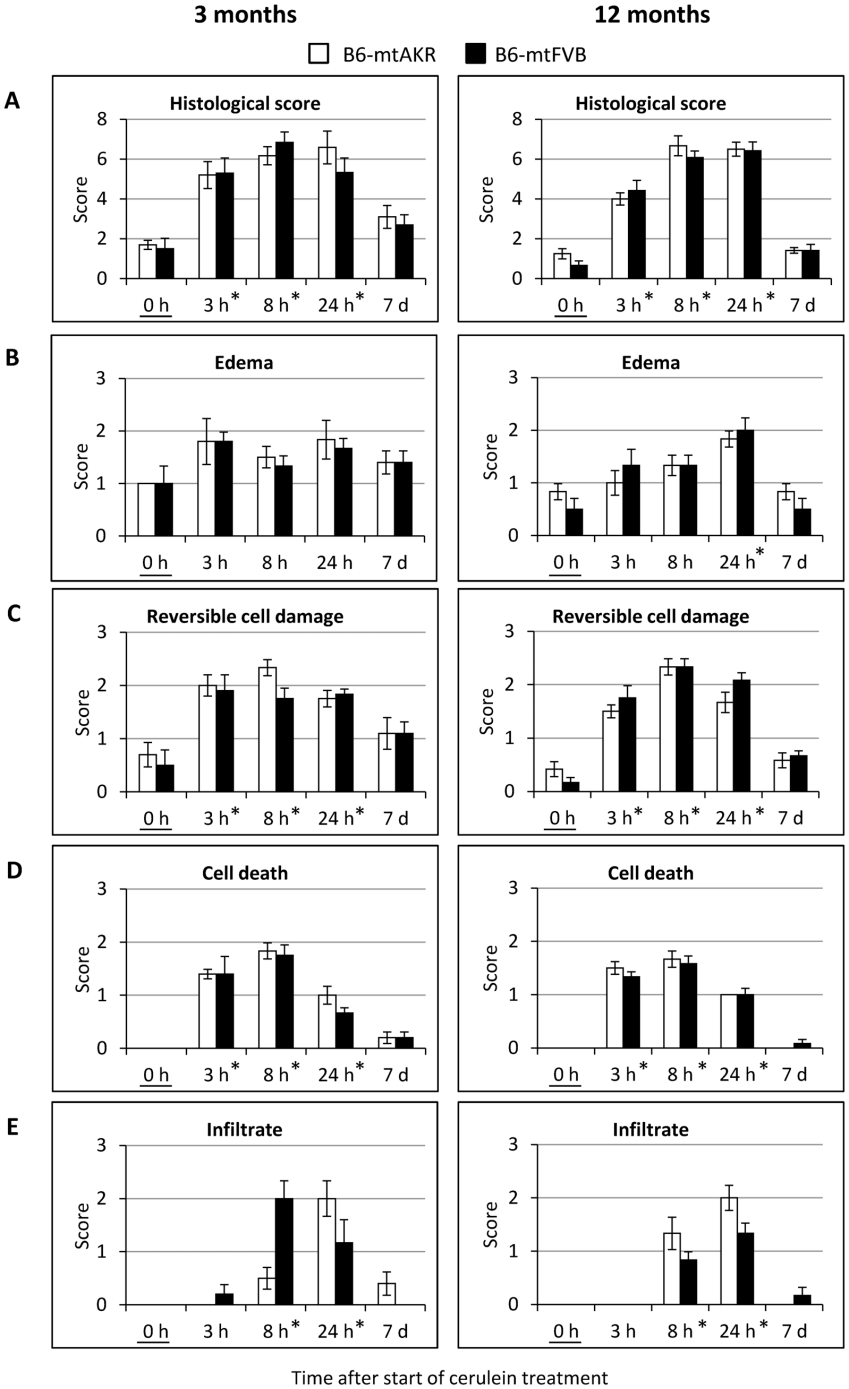
Evaluation of cerulein-induced pancreatic tissue damage in B6-mtAKR and B6-mtFVB mice. Three months and 12-month-old B6-mtAKR and B6-mtFVB mice were treated with cerulein as indicated (n = 5–6 per time point, strain and age group). Pancreatic sections of the mice were stained with H&E and scored as described in the “Materials and Methods” section. The total histological score shown in (A) represents the sum of the scores for edema (B), potentially reversible cell damage (C), cell death (D) and infiltration with immune cells (E), each of which scored on a scale from 0–3. Data are shown as mean ± SEM. *P_U_<0.005 vs. 0 h (control) with Bonferroni-adjusted α = 0.005.

For a more detailed analysis, histopathological changes were assessed by semiquantitative scores. In Fig. 2, the total histopathological score is shown in (A), while its components edema, reversible cell damage, cell death and inflammatory cell infiltrates are presented in (B–E). As expected, ANOVA revealed a statistically significant effect of the factor “time of cerulein treatment” on the total histopathological score (0 h vs. 3 h, 8 h and 24 h, P_Dunnett_<0.001 each). Furthermore, each of the 4 individual parameters shown in (B–E) was also significantly affected by the time of cerulein treatment (P_GLM Uni_<0.001 in each case). The effect of time within the two age groups is presented in Fig. 2 (A–E). In contrast, ANOVA did not indicate a significant effect of the factor “mouse strain” on the total histopathological score (A) as well as any of the individual parameters (B–E; P-values ranging between 0.342–0.742). Interestingly, however, significant effects of the factor “age” were observed: In the group of 3-month-old mice, higher scores for total histopathology (A) and edema (B) were detected (P_GLM Uni_ = 0.022 and 0.006, respectively; P-values C-E between 0.110–0.644).

**Figure 3:**
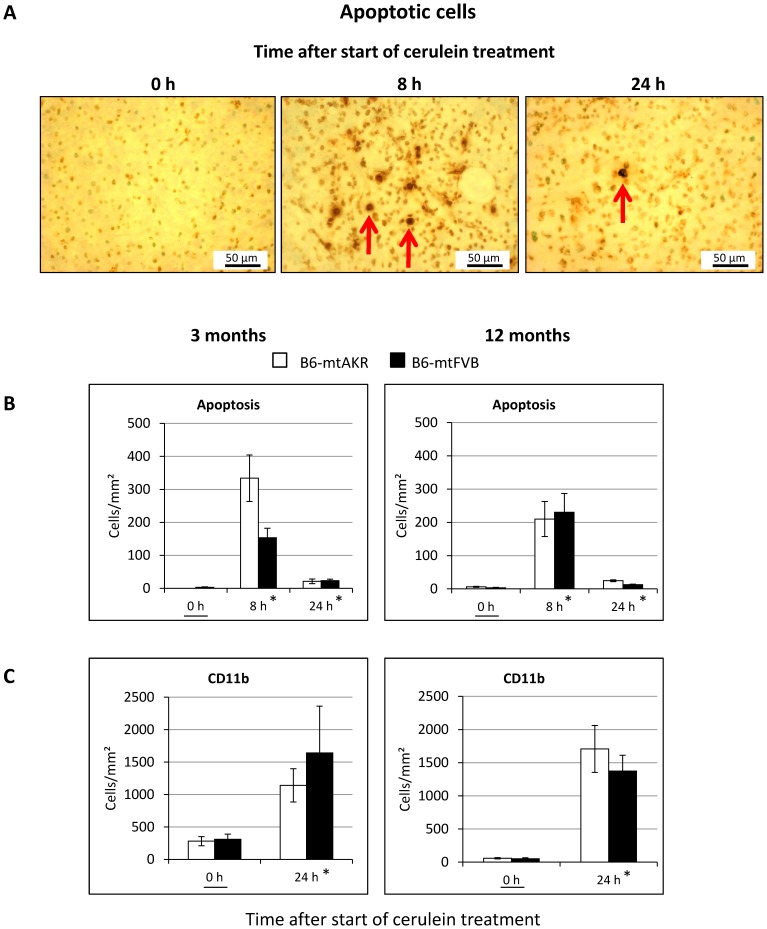
Detection of apoptotic cells and CD11b-positive cells in pancreatic tissue of B6-mtAKR and B6-mtFVB mice. Pancreatic sections of 3 and 12-month-old mice at the indicated time points after initiation of cerulein treatment (n = 5–6 per group) were stained with the ApopTag Kit for the detection of apoptotic cells (A, B), or processed applying the ABC technique and a CD11b-specific antibody (C). (A) Representative photographs of apoptotic cells (indicated by arrows) in the tissue of 12-month-old B6-mtFVB mice. (B, C) Positive-stained cells were counted, and mean values ± SEM were calculated. (B) *P_U_<0.0166 vs. 0 h with Bonferroni-adjusted α = 0.0166. (C) *P_U_<0.001 vs. 0 h each.

To further assess cell death in the course of cerulein-induced AP, apoptotic cells were detected based on the presence of DNA strand breaks. As shown in Fig. 3 A for the example of 12-month-old B6-mtFVB mice, large numbers of apoptotic cells were present in the pancreatic tissue at the time point 8 h, whereas they were largely absent in untreated animals as well as at 24 h after the start of cerulein treatment. A quantitative comparison of 3 and 12-month-old mice of both strains (Fig. 3 B) confirmed the expected significant effect of the factor “time of cerulein treatment” (0 h vs. 8 h, P_Dunnett_<0.001; for subgroup results see Fig. 3 B), whereas the mouse strain and the age of the animals did not show any significant influence on the number of apoptotic cells (P_GLM Uni_ = 0.180 and 0.703, respectively).

We have previously reported that inflammatory cell infiltrates in pancreatic tissue of cerulein-treated mice with a C57BL/6 background largely consist of CD11b-expressing cells (monocytes/macrophages, neutrophils, natural killer cells and granulocytes) [28]. For a quantitative assessment, we now chose the time points 0 h (untreated) and 24 h after the start of cerulein treatment (Fig. 3 C; for photographs, see section “Lymphocytic foci in B6-mtAKR and B6-mtFVB mice”). Application of cerulein was associated with a significant increase of CD11b-positive cells in pancreatic tissues (P_Kruskal-Wallis_<0.001, for subgroup results see Fig. 3 C), while there was no significant effect of the factors “mouse strain” and “age” (P = 0.774 and 0.333, respectively).

### Serum α-Amylase Levels and MPO Activity

**Figure 4:**
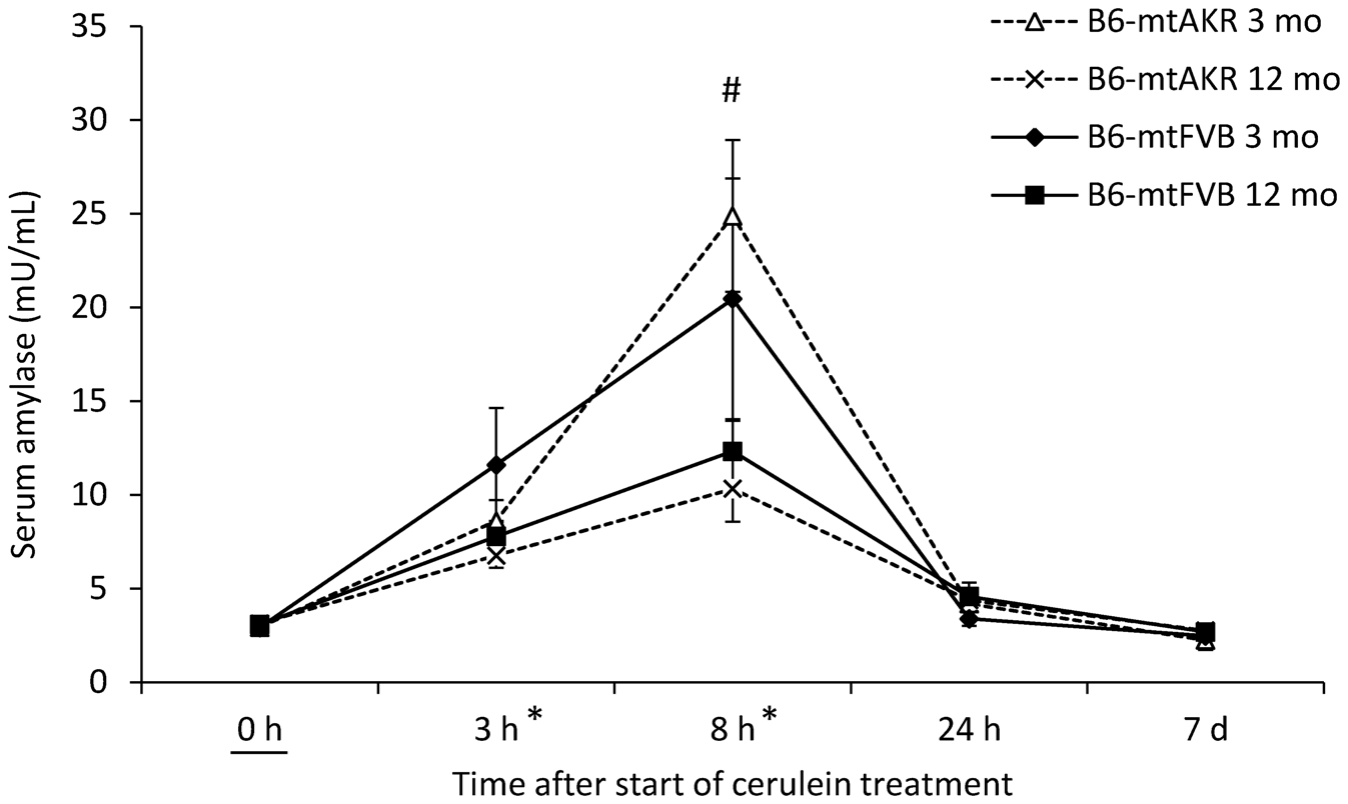
Measurement of α-amylase activities in B6-mtAKR and B6-mtFVB mice. Mice of two age groups (3 and 12 months, respectively; n = 5–6 per time point, strain and age group) were sacrificed at the indicated time points after the start of cerulein treatment and serum samples were subjected to the analysis of α-amylase activities. Data are expressed as units per mL of serum (mean values ± SEM). * P_Dunnett_<0.001 vs. 0 h; ^#^P_U_ = 0.014; 3 vs. 12-month-old mice.

A measurement of α-amylase activities in serum (Fig. 4) revealed the expected time-dependent increase in the course of cerulein-induced AP (0 h vs. 3 h and 8 h, P_Dunnett_<0.001 each). Again, there was no significant effect of the factor “mouse strain” (P_GLM Uni_ = 0.895), while the influence of the factor “age” was statistically significant (P_GLM Uni_ = 0.002). Subgroup-testing revealed higher levels of α-amylase in 3-month-old mice than in 12-month-old animals at the time point 8 h; the time of maximal α-amylase activity (P_U_ = 0.014).

**Figure 5:**
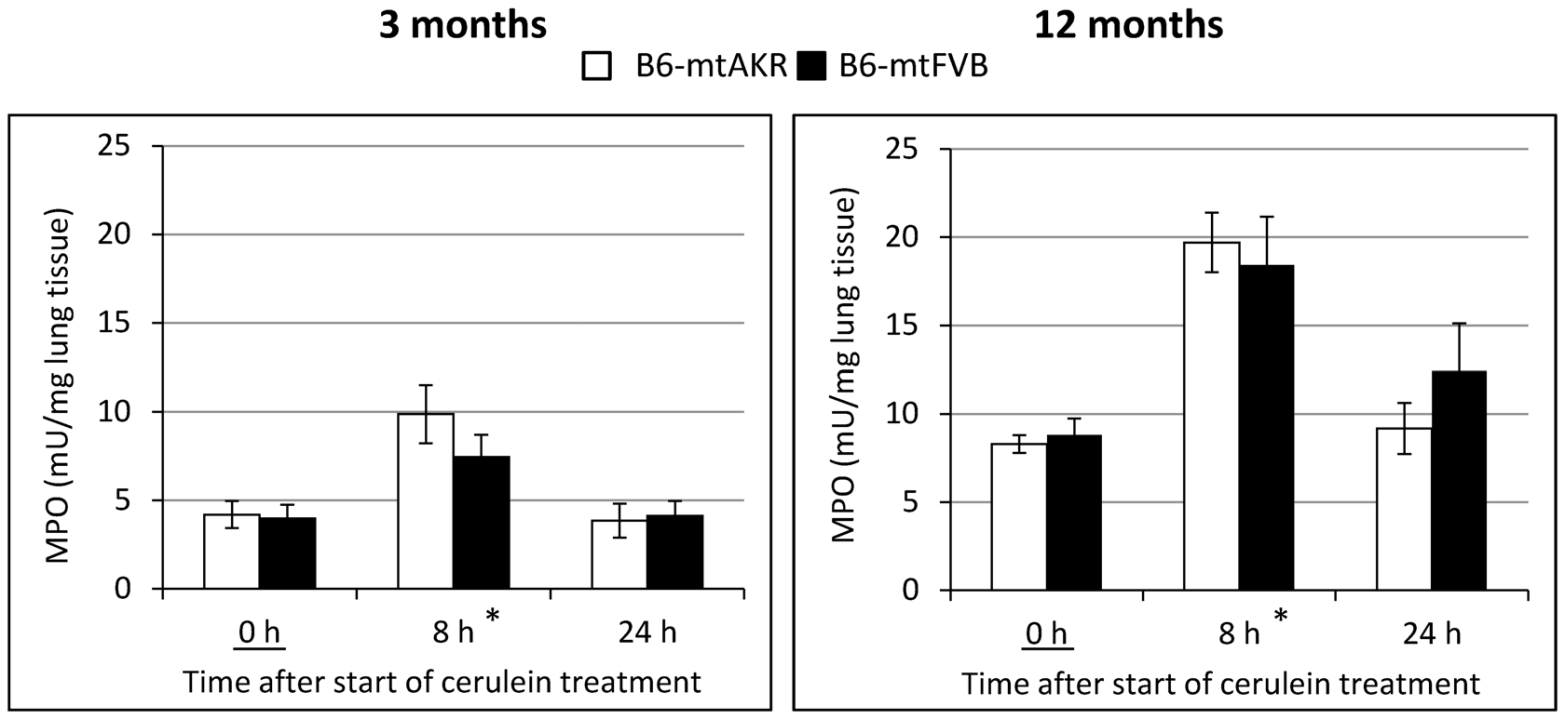
MPO activities in lung tissue of cerulein-treated B6-mtAKR and B6-mtFVB mice. Lung tissue of 3-month-old mice and 12-month-old animals at the indicated time points after initiation of cerulein treatment (n = 5–6 per per time point, strain and age group) was subjected to the quantification of MPO activity as described in the “Materials and Methods” section. Data are expressed as mU/mg wet weight (mean ± SEM). *P_U_ = 0.007 and <0.001, resp., vs. 0 h (Bonferroni-adjusted α = 0.0166).

As a surrogate marker of extrapancreatic inflammation related to AP, MPO activity in lung tissue of 3 and 12-month-old mice of both strains was determined (Fig. 5). Once more, multifactorial ANOVA revealed significant effects of the factors “time of cerulein treatment” (0 h vs. 8 h, P_Dunnett_<0.001) and “age” (P_GLM Uni_<0.001; higher values in 12-month-old mice), but not “mouse strain” (P_GLM Uni_ = 0.976). With respect to time and age dependencies, subgroup-testing indicated a significant increase of MPO activities at the time point 8 h, compared with untreated controls, in both age groups (3-month-old: P_U_ = 0.007; 12-month-old: <0.001). It should be noted, however, that increased MPO activities in the lung are not necessarily related to tissue damage since they may also reflect an increase of MPO-positive cells in the blood circulating through the lung vessels.

### Lymphocytic foci in B6-mtAKR and B6-mtFVB Mice

**Figure 6:**
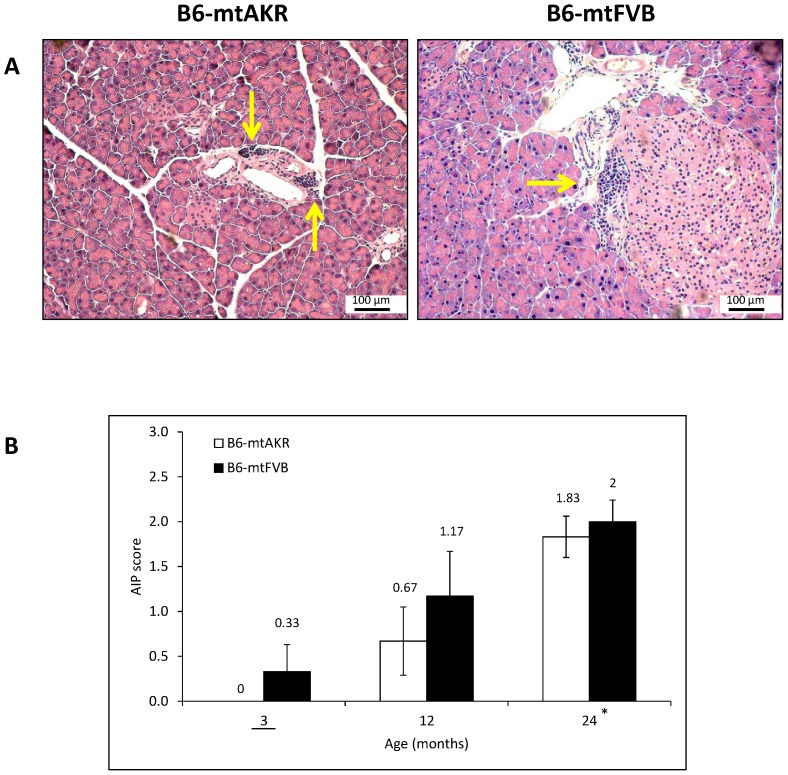
Foci of immune cells in B6-mtAKR and B6-mtFVB mice. Pancreatic sections of untreated 3, 12 and 24-month-old B6-mtAKR and B6-mtFVB mice were stained with H&E and analyzed regarding the presence of autoimmune-like pancreatic lesions. (A) The microphotographs show typical examples of immune cell infiltrates (indicated by arrows) with periductal, perivascular and periinsular location in 12-month-old B6-mtAKR (left) and B6-mtFVB (right) mice. (B) The samples were further evaluated by using a scoring system from 0 to 4. The data are indicated as mean ± SEM for n = 5–12 mice per experimental group. *P_U_<0.001 vs. 0 h (Bonferroni-adjusted α = 0.0166).

A careful evaluation of pancreatic tissue from 12-month-old untreated mice of both strains revealed the occasional presence of compact, usually small infiltrates of immune cells which surrounded ducts and vessels (Fig. 6 A) and were also found adjacent to pancreatic islets. The finding is in agreement with previous studies in aged mice [28], [32] and compatible with the early stage of autoimmune lesions as described before for other mouse strains [29], [30], [33]. To study if the appearance of immune cell infiltrates was indeed an age-dependent phenomenon, pancreatic tissue from three age groups (3, 12 and 24 months, respectively) was evaluated by using a scoring system (Fig. 6B). At an age of three months, autoimmune-like pancreatic lesions were absent in B6-mtAKR mice and rare in the B6-mtFVB strain. With increasing age, higher scores were determined in both strains. Analysis of variance confirmed that the factor “age” had a significant effect on disease severity (P_Kruskal-Wallis_<0.001, for subgroup-results see Fig. 6 B), but in contrast, the mouse strain had not (P_Kruskal-Wallis_ = 0.921).

**Figure 7:**
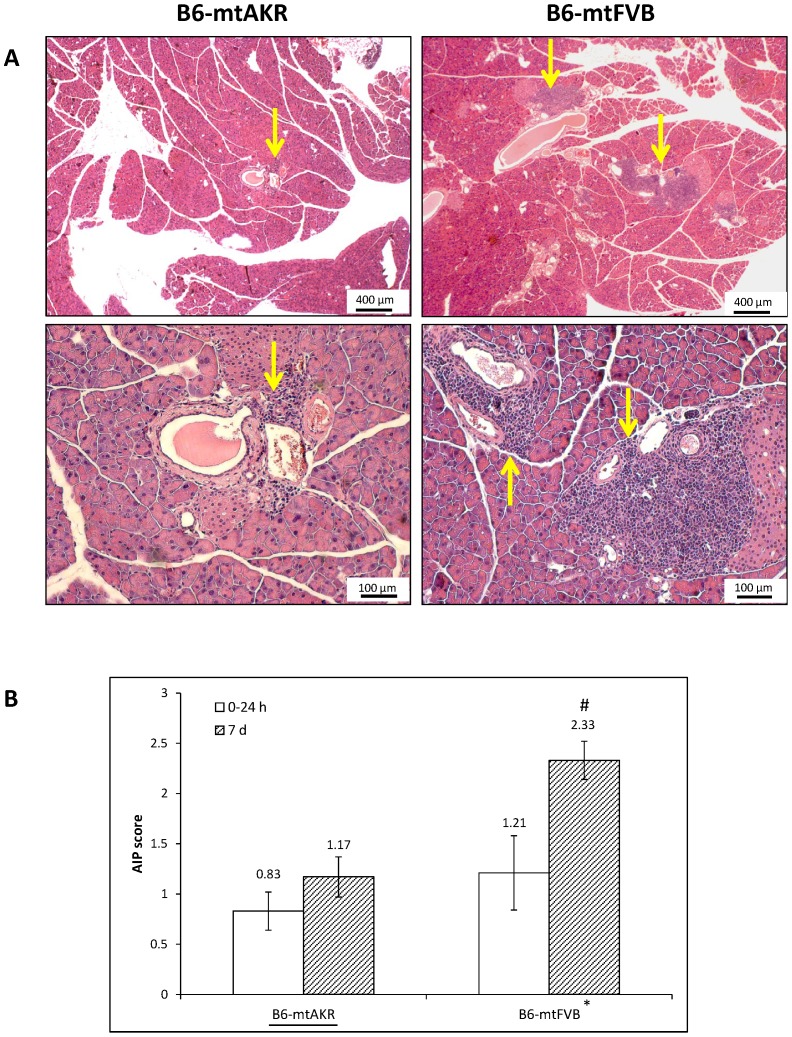
Effects of cerulein on the progression of autoimmune-like pancreatic lesions. Pancreatic sections of untreated and cerulein-treated 12-month-old B6-mtAKR and B6-mtFVB mice were stained with H&E and analyzed regarding the presence of autoimmune-like pancreatic lesions. (A) The microphotographs show typical examples of immune cell infiltrates, indicated by arrows, in B6-mtAKR (left photographs) and B6-mtFVB (right; the photographs show two different stains from the same animal) mice on day 7 after the start of cerulein treatment. (B) For further analysis, the mice of both strains were split into two groups, consisting of (*i*) all animals sacrificed at 0–24 h after the start of cerulein treatment (n = 24 per strain) and (*ii*) individuals of the day 7 group (n = 6 per strain). The results represent mean values ± SEM. * P_U_ = 0.046 (B6-mtAKR vs. B6-mtFVB mice), ^#^P_U_ = 0.021 (0–24 h vs. day 7 mice).

We next studied how application of cerulein affected the progression of autoimmune-like pancreatic lesions. In 3-month-old mice of both strains, AIP foci remained very rare or absent. Furthermore, 12-month-old mice at the time points 3, 8 and 24 h after the start of cerulein treatment were indistinguishable from untreated mice with respect to the frequency and size of immune cell infiltrates (for data, see “Supporting Information”, Data S1). On day 7 however, when the repair of cerulein-induced tissue damage was largely completed (Fig. 1 E), in B6-mtFVB mice, but not in B6-mtAKR mice, a significantly increased score compared to mice sacrificed at 0–24 h after the start of cerulein treatment was determined (Fig. 7; P_U_ = 0.021), suggesting aggravation of autoimmune-like lesions. Specifically, enlarged foci of immune cells and a somewhat more pronounced replacement of exocrine tissue were observed. Furthermore, statistical analysis showed that the factor “mouse strain”, under the new conditions, indeed had a significant effect on the formation of immune cell infiltrates (n = 30 per strain, P_U_ = 0.046). Still, it has to be noted that autoimmune-like pancreatic lesions of B6-mtFVB mice remained relatively mild and affected a minor portion of the exocrine pancreas only.

**Figure 8:**
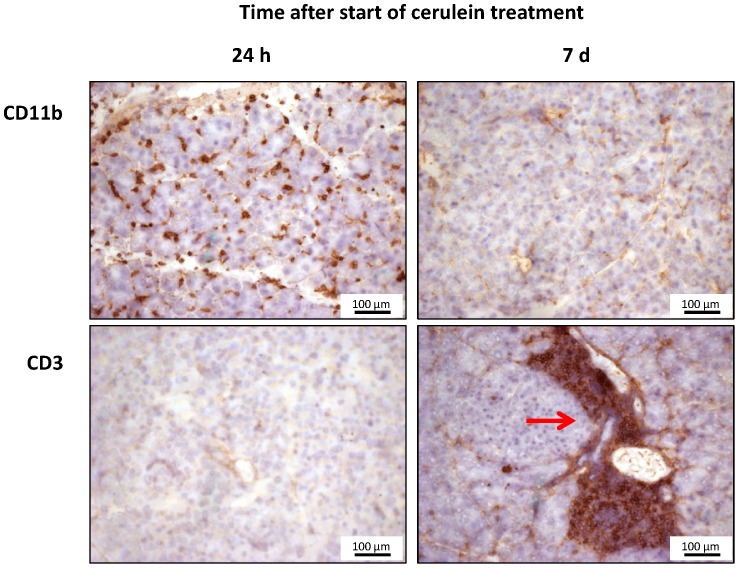
Detection of CD11b-positive and CD3-expressing cells in pancreatic tissue. All samples are from 12-month-old B6-mtFVB mice but are representative for animals of both strains. Pancreatic sections of mice at the indicated time points after the start of cerulein treatment were stained with anti-CD11b and anti-CD3 antibodies, respectively. Upper row: large numbers of scattered CD11b-positive cells at 24 h (left), only background staining on day 7 (right). Lower row: 24 h (left) – faint background staining only in a selected region without autoimmune-like pancreatic lesions; day 7 (right): focal infiltrate of inflammatory cells, many of them CD3-positive; background staining of the surrounding pancreatic tissue.

To further distinguish between age-associated foci of immune cells and cerulein-induced transient inflammation, cell infiltrates in pancreatic tissue were characterized by immunohistochemistry (Fig. 8). 24 h after the start of cerulein treatment, CD11b-positive cells were present in large numbers and showed a scattered pattern of distribution (for quantification, see Fig. 3 C). On day 7, only very few CD11b-positive cells were still detectable in the tissue. The foci of immune cells, on the other hand, largely consisted of CD3-positive lymphocytes, which were otherwise very rare both at 24 h and 7 d after the induction of experimental AP.

### *In vitro* Studies with Pancreatic Acini

**Figure 9:**
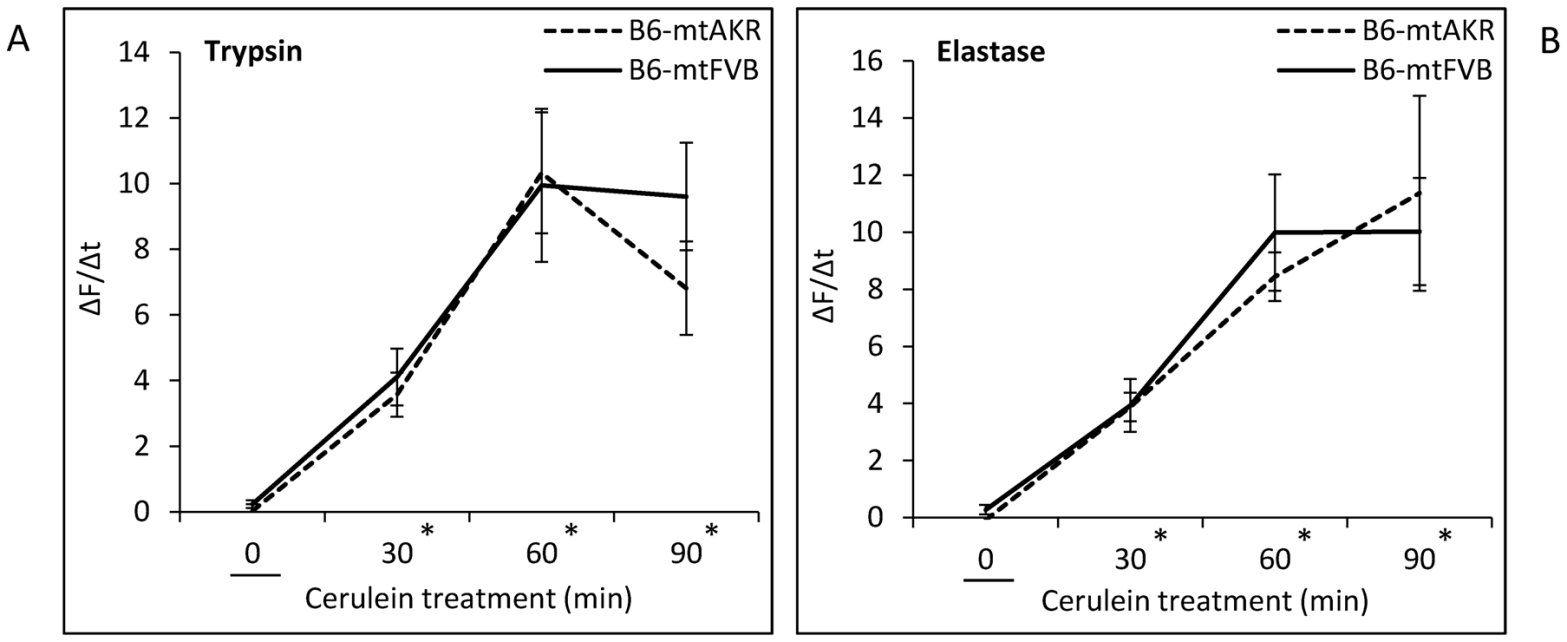
Trypsin and elastase activities in pancreatic acini isolated from B6-mtAKR and B6-mtFVB mice. Isolated pancreatic acini of 12-month-old mice of the two strains were treated *in vitro* with cerulein (10 nmol/L) for up to 90 min. Subsequently, cleavage of rhodamine 110-labeled synthetic substrates for (A) trypsin and (B) elastase was recorded by cytofluorometry. To compare proteolytic activities, ΔF/Δt ratios were calculated and data expressed as (ΔF/Δt)_cerulein_-(ΔF/Δt)_untreated_ (mean ± SEM, n = 6 per experimental group). * P_Simple Contrast_≤0.001 vs. 0 min each.

**Figure 10:**
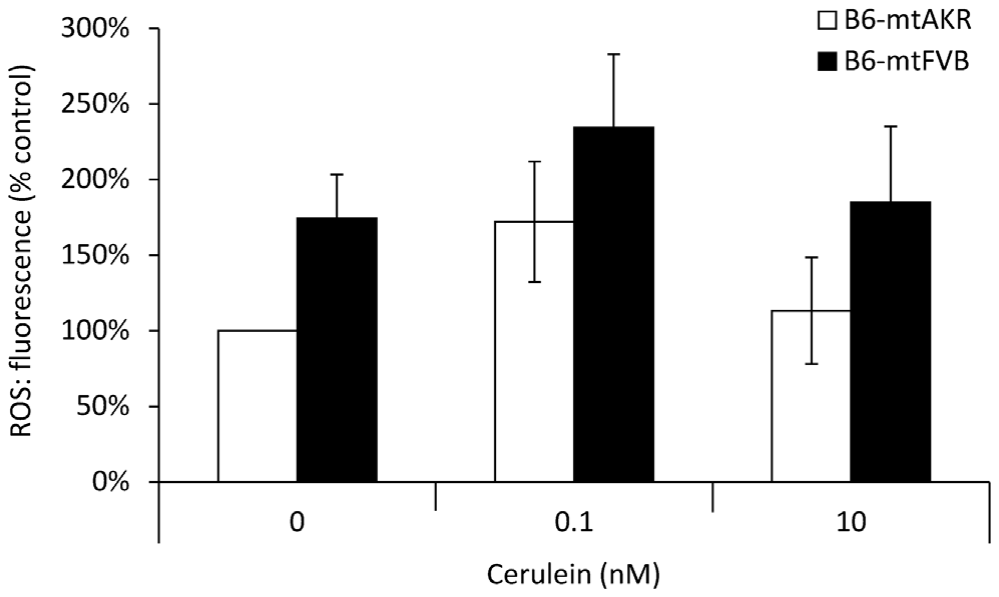
ROS levels in pancreatic acini isolated from B6-mtAKR and B6-mtFVB mice. DCFH-DA-labeled pancreatic acini of 12-month-old mice of the two strains were treated *in vitro* with cerulein at the indicated concentrations for 30 min before fluorescence intensity of 2′,7′-dichlorofluorescein was recorded. Data of 6 independent samples per experimental protocol were used to calculate mean values ± SEM.

To further study the functional consequences of the mtDNA nt7778 G/T polymorphism, *in vitro* experiments were performed. Therefore, pancreatic acini from 12-month-old B6-mtAKR and B6-mtFVB mice were challenged with cerulein, and intracellular activation of pancreatic proteases was monitored (Fig. 9). As expected, a rapid increase of both trypsin (9 A) and elastase (9 B) activities in response to cerulein treatment was observed (factor “time”: P_GLM Rep_<0.001 for trypsin and elastase as well, for contrast-testing see Fig. 9). Significant differences between the two strains, however, were neither detected for trypsin (P = 0.596) nor for elastase (P = 0.916). Furthermore, measurements of the intraacinar ROS levels indicated tendencies to higher concentrations in cells of the B6-mtFVB strain as well as in cerulein-treated acini of both strains. In our samples, however, statistical significance could not be shown (Fig. 10).

## Discussion

Mitochondrial gene mutations are increasingly acknowledged as risk factors for the development of human diseases. With respect to the mitochondrial *Atp8* gene, the nucleotide transition nt8406 C/T has been shown to be associated with multiple sclerosis [34], whereas the mutation nt8529 G/A results in a defective mitochondrial oxidative phosphorylation with cardiomyopathy and neuropathy [35]. Here we have addressed the question if the murine mtDNA polymorphism nt7778 G/T affects the susceptibility to develop severe AP upon challenge with the standard trigger cerulein. The investigations were motivated on one hand by the established role of mitochondrial dysfunction in the pathogenesis of AP [4]–[10] and on the other hand by previous studies in conplastic mouse strains that had suggested, in addition to other biochemical and cellular abnormalities [19], [20], [22], [23], an increased incidence of autoimmune chronic pancreatitis in mice carrying the nt7778 G/T polymorphism [19]. The results of our studies in conplastic mouse strains of two age groups suggest that the time course and the severity of cerulein-induced AP are not affected by the nt7778 G/T polymorphism. However, strain-specific differences in the progression of autoimmune-like pancreatic lesions, which were histologically clearly distinguishable from cerulein-induced AP, were detected: Our results indicate that treatment of B6-mtFVB but not the control mice with cerulein induced a significant augmentation of lymphocytic foci within 7 days of the application of the drug. We conclude from this observation that, in the context of an autoimmune-prone background, additional tissue damage may cause an exacerbation of an autoimmune disease. We hypothesize that the release of tissue antigens enhances the activation of autoreactive T cells, which in turn trigger the progression of pancreatic lesions. It has to be noted, however, that no extended tissue damage, as it is typical for advanced chronic AIP, was observed. Due to this limitation, we have preferred the term “autoimmune-like pancreatic lesions” to describe the histopathological changes which were detected.

The lack of any effect of the nt7778 G/T polymorphism on the severity of cerulein-induced AP was somewhat unexpected, since (1) previous studies had shown increased oxidative stress in cells and tissues of B6-mtFVB mice [19], [20], [22] and (2) various links between ROS and the pathogenesis of AP have been suggested [11]–[14]. To gain further mechanistic insights, isolated pancreatic acinar cells were challenged with cerulein *in vitro* as previously described [28]. These studies, however, did not reveal significant differences between the two strains regarding intracellular ROS levels and activation of the proteases trypsin and elastase, independent of the presence or absence of cerulein.

Together, our data suggest that the nt7778 G/T polymorphism has no effect on pancreatic acinar cell functions both under physiological and pathophysiological conditions. Along with previous studies of our group [28], the results also point to effective mechanisms of antioxidative defense in pancreatic acinar cells, which may compensate for increased ROS production in cells with genetic defects or relevant gene polymorphisms.

The results of this study are in line with previous reports showing that mutations and variations in mitochondrial or nuclear genes encoding mitochondrial proteins can be associated with experimental autoimmune diseases [18], [36]–[40]. How precisely the mtDNA polymorphism nt7778 G/T augments formation of lymphocytic foci in the context of additional tissue damage remains to be studied further. According to a plausible hypothesis, increased mitochondrial ROS levels in T cells may activate cytoprotective gene transcription and thus support cell survival under hypoxic conditions in inflamed tissues [19]. Together with a cerulein-induced release of pancreatic antigens, this may eventually lead to the progression of lymphocytic lesions in mice carrying the mitochondrial *Atp8* polymorphism.

An additional finding of this study is the age-dependent difference in the response of mice to the treatment with cerulein. Specifically, we found that maximum α-amylase activities (Fig. 4) and also the pancreatic histopathological score and the degree of edema (Fig. 2) decreased with age, while MPO activity in lung tissue (Fig. 5) was higher in the older mice. No significant age effect was observed for apoptotic cell death and the number of infiltrating CD11b-positive inflammatory cells (Fig. 3). With respect to α-amylase, our data are in agreement with a previous study by Okamura *et al.* [41] who also detected (at a time point comparable to 8 h in this study) higher enzyme activities in sera of young mice. On the other hand, Okamura *et al.* [41] observed (in analogy to clinical studies) an increased severity of AP in old mice (23–25 months). While our finding of an increased MPO activity in aged mice points into the same direction, the lower pancreatic histopathological score appears to be contradictory. It has to be noted, however, that the aged mice that were used in this study were much younger than those studied by Okamura *et al.* [41] (12 vs. 23–25 months), and should therefore be considered as middle-aged only.

The molecular mechanisms that underlie age-dependent differences in the response to pancreatic injury are still largely unknown. One important aspect to study will be the role of phosphatidylinositol 3-kinase/AKT (protein kinase B) signaling, since this signaling pathway has previously been implicated in the diminished pancreatic regeneration of old mice after partial pancreatic resection as well as an age-dependent loss of the responsiveness of pancreatic acinar cells to growth factors [42], [43].

## Supporting Information

Data S1 **Raw data of the** ***in vivo*** **and in** ***vitro*** **investigations.** The data are structured as follows: Pages 1–8 provide detailed results for individual mice that were employed in the cerulein experiments. On page 9, the scores for autoimmune-like pancreatic lesions of 24-month-old are shown. The results of trypsin, elastase and ROS measurements *in vitro* are presented on page 10.
(PDF)
