# Supplementary material for: The mtDNA nt7778 G/T Polymorphism Augments Formation of Lymphocytic Foci but Does Not Aggravate Cerulein-Induced Acute Pancreatitis in Mice
Source: PLoS One. 2014 Jul 10;9(7):e102266. doi: 10.1371/journal.pone.0102266 (PMC4092110; doi:10.1371/journal.pone.0102266)
Supplement: Data S1 — Raw data of the in vivo and in vitro investigations. The data are structured as follows: Pages 1–8 provide detailed results for individual mice that were employed in the cerulein experiments. On page 9, the scores for autoimmune-like pancreatic lesions of 24-month-old are shown. The results of trypsin, elastase and ROS measurements in vitro are presented on page 10. (PDF) [file pone.0102266.s001.pdf]

3-months-old mice

Mouse strain	Time of cerulein treatment	No.	Sex	Weight (g)	Scores cerulein pancreatitis: Fig. 2				
					Histo-Score (2A)	Edema (2B)	Reversible damage (2C)	Cell death (2D)	Infiltrates (2 E)
B6-mtAKR	0 h	1	m	26.4	2.0	1.0	1.0	0.0	0.0
B6-mtAKR	0 h	2	m	24.9	1.5	1.0	0.5	0.0	0.0
B6-mtAKR	0 h	3	f	24.9	2.5	1.0	1.5	0.0	0.0
B6-mtAKR	0 h	4	f	18.8	1.0	1.0	0.0	0.0	0.0
B6-mtAKR	0 h	5	m	27.2	1.5	1.0	0.5	0.0	0.0
B6-mtAKR	3 h	1	m	26.6	7.0	3.0	2.5	1.5	0.0
B6-mtAKR	3 h	2	f	20.1	4.5	1.0	2.0	1.5	0.0
B6-mtAKR	3 h	3	f	19.8	4.0	1.0	1.5	1.5	0.0
B6-mtAKR	3 h	4	m	22.8	7.0	3.0	2.5	1.5	0.0
B6-mtAKR	3 h	5	f	20.6	3.5	1.0	1.5	1.0	0.0
B6-mtAKR	8 h	1	m	30.5	5.5	2.0	2.0	1.5	0.0
B6-mtAKR	8 h	2	m	28.3	6.0	1.0	2.0	2.0	1.0
B6-mtAKR	8 h	3	f	21.7	7.0	2.0	2.5	1.5	1.0
B6-mtAKR	8 h	4	f	21.5	6.0	1.0	2.5	2.5	0.0
B6-mtAKR	8 h	5	m	27.3	8.0	2.0	3.0	2.0	1.0
B6-mtAKR	8 h	6	f	21	4.5	1.0	2.0	1.5	0.0
B6-mtAKR	24 h	1	m	29.5	4.5	1.0	1.0	0.5	2.0
B6-mtAKR	24 h	2	f	21.4	4.5	1.0	1.5	1.0	1.0
B6-mtAKR	24 h	3	f	21.9	9.0	3.0	2.0	1.0	3.0
B6-mtAKR	24 h	4	m	33.2	5.5	1.0	2.0	1.5	1.0
B6-mtAKR	24 h	5	f	20.2	6.5	2.0	2.0	0.5	2.0
B6-mtAKR	24 h	6	m	28.1	9.5	3.0	2.0	1.5	3.0
B6-mtAKR	7 d	1	m	32.4	5.5	2.0	2.0	0.5	1.0
B6-mtAKR	7 d	2	f	21	3.0	1.0	1.5	0.5	0.0
B6-mtAKR	7 d	3	m	30.2	2.0	1.0	1.0	0.0	0.0
B6-mtAKR	7 d	4	m	26.9	2.0	1.0	1.0	0.0	0.0
B6-mtAKR	7 d	5	f	20.4	3.0	2.0	0.0	0.0	1.0

3-months-old mice

Mouse strain	Time of cerulein treatment	No.	Sex	Weight (g)	Scores cerulein pancreatitis: Fig. 2				
					Histo-Score (2A)	Edema (2B)	Reversible damage (2C)	Cell death (2D)	Infiltrates (2 E)
B6-mtFVB	0 h	1	m	29.7	0.5	0.0	0.5	0.0	0.0
B6-mtFVB	0 h	2	f	22.3	2.0	2.0	0.0	0.0	0.0
B6-mtFVB	0 h	3	f	19	1.5	1.0	0.5	0.0	0.0
B6-mtFVB	0 h	4	m	26.8	0.0	0.0	0.0	0.0	0.0
B6-mtFVB	0 h	5	f	20	4.0	2.0	2.0	0.0	0.0
B6-mtFVB	0 h	6	m	25.9	1.0	1.0	0.0	0.0	0.0
B6-mtFVB	3 h	1	m	30.6	3.5	2.0	1.0	0.5	0.0
B6-mtFVB	3 h	2	f	18.8	3.5	1.0	1.5	1.0	0.0
B6-mtFVB	3 h	3	m	21.8	7.5	2.0	3.0	2.5	0.0
B6-mtFVB	3 h	4	f	20.7	5.0	2.0	2.0	1.0	0.0
B6-mtFVB	3 h	5	m	28	7.0	2.0	2.0	2.0	1.0
B6-mtFVB	8 h	1	m	27.6	5.5	1.0	1.0	2.5	1.0
B6-mtFVB	8 h	2	f	21.8	5.0	1.0	1.5	1.5	1.0
B6-mtFVB	8 h	3	f	20.3	8.5	1.0	2.5	2.0	3.0
B6-mtFVB	8 h	4	m	26.4	8.0	1.0	2.0	2.0	3.0
B6-mtFVB	8 h	5	f	20	7.5	2.0	2.0	1.5	2.0
B6-mtFVB	8 h	6	m	25.5	6.5	2.0	1.5	1.0	2.0
B6-mtFVB	24 h	1	f	20.1	3.5	1.0	2.0	0.5	0.0
B6-mtFVB	24 h	2	f	20.4	3.0	1.0	1.5	0.5	0.0
B6-mtFVB	24 h	3	m	25.4	5.5	2.0	2.0	0.5	1.0
B6-mtFVB	24 h	4	m	31.1	5.0	2.0	1.5	0.5	1.0
B6-mtFVB	24 h	5	f	19.6	7.0	2.0	2.0	1.0	2.0
B6-mtFVB	24 h	6	f	20.7	8.0	2.0	2.0	1.0	3.0
B6-mtFVB	7 d	1	f	21.7	1.5	1.0	0.5	0.0	0.0
B6-mtFVB	7 d	2	f	19.8	4.0	2.0	1.5	0.5	0.0
B6-mtFVB	7 d	3	m	22.8	4.0	2.0	1.5	0.5	0.0
B6-mtFVB	7 d	4	f	17.9	1.5	1.0	0.5	0.0	0.0
B6-mtFVB	7 d	5	m	25.6	2.5	1.0	1.5	0.0	0.0

3-months-old mice

Mouse strain	Time of cerulein treatment	No.	Sex	Weight (g)	Amylase (U/mL)	MPO (mU/mg lung)	ApopTag (cells/mm²)	CD11b (cells/mm²)
					Fig. 4	Fig. 5	Fig. 3	Fig. 3
B6-mtAKR	0 h	1	m	26.4	2.76	4.22	0.00	204.80
B6-mtAKR	0 h	2	m	24.9	2.80	5.75	0.00	147.20
B6-mtAKR	0 h	3	f	24.9	2.98	6.34	0.00	435.20
B6-mtAKR	0 h	4	f	18.8	2.78	2.81	0.00	115.20
B6-mtAKR	0 h	5	m	27.2	3.36	1.82	0.00	502.40
B6-mtAKR	3 h	1	m	26.6	9.27			
B6-mtAKR	3 h	2	f	20.1	5.59			
B6-mtAKR	3 h	3	f	19.8	9.45			
B6-mtAKR	3 h	4	m	22.8	11.90			
B6-mtAKR	3 h	5	f	20.6	6.78			
B6-mtAKR	8 h	1	m	30.5	29.80	10.28	307.20	
B6-mtAKR	8 h	2	m	28.3	20.96	14.58	390.40	
B6-mtAKR	8 h	3	f	21.7	39.77	12.84	672.00	
B6-mtAKR	8 h	4	f	21.5	11.04	2.15	288.00	
B6-mtAKR	8 h	5	m	27.3	19.46	7.96	115.20	
B6-mtAKR	8 h	6	f	21	28.23	11.36	230.40	
B6-mtAKR	24 h	1	m	29.5	4.36	7.95	19.20	960.00
B6-mtAKR	24 h	2	f	21.4	5.17	1.90	12.80	1478.40
B6-mtAKR	24 h	3	f	21.9	2.76	2.36	19.20	294.40
B6-mtAKR	24 h	4	m	33.2	3.57	1.33	57.60	947.20
B6-mtAKR	24 h	5	f	20.2	3.65	3.67	12.80	854.40
B6-mtAKR	24 h	6	m	28.1	5.57	5.87	6.40	2310.40
B6-mtAKR	7 d	1	m	32.4	3.06			
B6-mtAKR	7 d	2	f	21	0.24			
B6-mtAKR	7 d	3	m	30.2	2.78			
B6-mtAKR	7 d	4	m	26.9	2.93			
B6-mtAKR	7 d	5	f	20.4	2.21			

3-months-old mice

Mouse strain	Time of cerulein treatment	No.	Sex	Weight (g)	Amylase (U/mL)	MPO (mU/mg lung)	ApopTag (cells/mm²)	CD11b (cells/mm²)
					Fig. 4	Fig. 5	Fig. 3	Fig. 3
B6-mtFVB	0 h	1	m	29.7	2.86	6.26	6.40	83.20
B6-mtFVB	0 h	2	f	22.3	2.69	5.43	0.00	236.80
B6-mtFVB	0 h	3	f	19	4.61	5.34	6.40	140.80
B6-mtFVB	0 h	4	m	26.8	2.23	0.93	0.00	275.20
B6-mtFVB	0 h	5	f	20	2.51	2.29	0.00	659.20
B6-mtFVB	0 h	6	m	25.9	2.60	3.63	6.40	454.40
B6-mtFVB	3 h	1	m	30.6	19.35			
B6-mtFVB	3 h	2	f	18.8	8.94			
B6-mtFVB	3 h	3	m	21.8	12.70			
B6-mtFVB	3 h	4	f	20.7	7.08			
B6-mtFVB	3 h	5	m	28	9.86			
B6-mtFVB	8 h	1	m	27.6	27.78	12.43	153.60	
B6-mtFVB	8 h	2	f	21.8	11.84	7.74	147.20	
B6-mtFVB	8 h	3	f	20.3	7.37	5.49	185.60	
B6-mtFVB	8 h	4	m	26.4	34.41	4.99	243.20	
B6-mtFVB	8 h	5	f	20	6.99	3.74	12.80	
B6-mtFVB	8 h	6	m	25.5	34.28	10.30	179.20	
B6-mtFVB	24 h	1	f	20.1	4.32	4.92	12.80	326.40
B6-mtFVB	24 h	2	f	20.4	3.38	5.48	38.40	1004.80
B6-mtFVB	24 h	3	m	25.4	3.82	7.50	32.00	512.00
B6-mtFVB	24 h	4	m	31.1	1.94	1.84	12.80	710.40
B6-mtFVB	24 h	5	f	19.6	2.75	2.72	12.80	1875.20
B6-mtFVB	24 h	6	f	20.7	4.06	2.26	32.00	5420.80
B6-mtFVB	7 d	1	f	21.7	2.40			
B6-mtFVB	7 d	2	f	19.8	2.19			
B6-mtFVB	7 d	3	m	22.8	2.50			
B6-mtFVB	7 d	4	f	17.9	2.38			
B6-mtFVB	7 d	5	m	25.6	2.87			

12-months-old mice

Mouse strain	Time of cerulein treatment	No.	Sex	Weight	Scores cerulein pancreatitis: Fig. 2				
					Histo-Score (2A)	Edema (2B)	Reversible damage (2C)	Cell death (2D)	Infiltrates (2 E)
B6-mtAKR	0 h	1	m	28.5	1.0	1.0	0.0	0.0	0.0
B6-mtAKR	0 h	2	m	34.6	2.0	1.0	1.0	0.0	0.0
B6-mtAKR	0 h	3	f	25.2	1.5	1.0	0.5	0.0	0.0
B6-mtAKR	0 h	4	m	38.4	1.5	1.0	0.5	0.0	0.0
B6-mtAKR	0 h	5	f	30.1	1.5	1.0	0.5	0.0	0.0
B6-mtAKR	0 h	6	f	26.4	0.0	0.0	0.0	0.0	0.0
B6-mtAKR	3 h	1	m	36.2	5.0	2.0	1.5	1.5	0.0
B6-mtAKR	3 h	2	m	37.7	4.0	1.0	2.0	1.0	0.0
B6-mtAKR	3 h	3	m	37.1	4.0	1.0	1.5	1.5	0.0
B6-mtAKR	3 h	4	m	35.0	4.0	1.0	1.5	1.5	0.0
B6-mtAKR	3 h	5	f	37.2	4.5	1.0	1.5	2.0	0.0
B6-mtAKR	3 h	6	f	30.1	2.5	0.0	1.0	1.5	0.0
B6-mtAKR	8 h	1	m	44.0	7.0	2.0	2.0	2.0	1.0
B6-mtAKR	8 h	2	m	39.9	5.5	1.0	2.5	1.0	1.0
B6-mtAKR	8 h	3	f	26.5	9.0	1.0	3.0	2.0	3.0
B6-mtAKR	8 h	4	f	30.4	5.5	1.0	2.0	1.5	1.0
B6-mtAKR	8 h	5	m	31.2	7.0	2.0	2.5	1.5	1.0
B6-mtAKR	8 h	6	f	35.0	6.0	1.0	2.0	2.0	1.0
B6-mtAKR	24 h	1	f	35.4	7.5	2.0	1.5	1.0	3.0
B6-mtAKR	24 h	2	m	42.4	7.5	2.0	2.5	1.0	2.0
B6-mtAKR	24 h	3	f	25.4	6.0	2.0	1.0	1.0	2.0
B6-mtAKR	24 h	4	f	29.6	5.5	2.0	1.5	1.0	1.0
B6-mtAKR	24 h	5	m	34.4	7.0	2.0	2.0	1.0	2.0
B6-mtAKR	24 h	6	m	36.1	5.5	1.0	1.5	1.0	2.0
B6-mtAKR	7 d	1	m	39.0	1.5	1.0	0.5	0.0	0.0
B6-mtAKR	7 d	2	m	40.2	2.0	1.0	1.0	0.0	0.0
B6-mtAKR	7 d	3	m	39.4	1.0	0.0	1.0	0.0	0.0
B6-mtAKR	7 d	4	f	24.5	1.0	1.0	0.0	0.0	0.0
B6-mtAKR	7 d	5	f	23.8	1.5	1.0	0.5	0.0	0.0
B6-mtAKR	7 d	6	f	24.8	1.5	1.0	0.5	0.0	0.0

12-months-old mice

Mouse strain	Time of cerulein treatment	No.	Sex	Weight	Scores cerulein pancreatitis: Fig. 2				
					Histo-Score (2A)	Edema (2B)	Reversible damage (2C)	Cell death (2D)	Infiltrates (2 E)
B6-mtFVB	0 h	1	f	26.0	0.0	0.0	0.0	0.0	0.0
B6-mtFVB	0 h	2	m	38.2	0.5	0.0	0.5	0.0	0.0
B6-mtFVB	0 h	3	f	26.8	0.0	0.0	0.0	0.0	0.0
B6-mtFVB	0 h	4	m	35.5	1.0	1.0	0.0	0.0	0.0
B6-mtFVB	0 h	5	f	32.3	1.5	1.0	0.5	0.0	0.0
B6-mtFVB	0 h	6	m	41.5	1.0	1.0	0.0	0.0	0.0
B6-mtFVB	3 h	1	f	31.9	3.0	1.0	1.0	1.0	0.0
B6-mtFVB	3 h	2	m	35.0	7.0	3.0	2.5	1.5	0.0
B6-mtFVB	3 h	3	f	35.5	4.5	1.0	2.0	1.5	0.0
B6-mtFVB	3 h	4	m	38.3	3.5	1.0	1.0	1.5	0.0
B6-mtFVB	3 h	5	f	28.9	4.0	1.0	2.0	1.0	0.0
B6-mtFVB	3 h	6	m	42.1	4.5	1.0	2.0	1.5	0.0
B6-mtFVB	8 h	1	f	34.2	5.5	1.0	2.0	1.5	1.0
B6-mtFVB	8 h	2	f	33.9	5.0	1.0	2.0	1.0	1.0
B6-mtFVB	8 h	3	f	36.4	6.5	1.0	3.0	1.5	1.0
B6-mtFVB	8 h	4	f	38.3	7.0	2.0	2.5	1.5	1.0
B6-mtFVB	8 h	5	f	42.1	5.5	1.0	2.5	2.0	0.0
B6-mtFVB	8 h	6	m	40.8	7.0	2.0	2.0	2.0	1.0
B6-mtFVB	24 h	1	f	27.5	6.0	2.0	2.0	1.0	1.0
B6-mtFVB	24 h	2	f	28.3	7.0	2.0	2.5	1.5	1.0
B6-mtFVB	24 h	3	m	37.2	8.0	3.0	2.5	0.5	2.0
B6-mtFVB	24 h	4	m	38.4	6.0	2.0	2.0	1.0	1.0
B6-mtFVB	24 h	5	f	24.7	7.0	2.0	2.0	1.0	2.0
B6-mtFVB	24 h	6	m	42.7	4.5	1.0	1.5	1.0	1.0
B6-mtFVB	7 d	1	m	36.2	1.5	1.0	0.5	0.0	0.0
B6-mtFVB	7 d	2	m	41.5	0.5	0.0	0.5	0.0	0.0
B6-mtFVB	7 d	3	f	32.7	2.5	1.0	1.0	0.5	0.0
B6-mtFVB	7 d	4	f	42.9	0.5	0.0	0.5	0.0	0.0
B6-mtFVB	7 d	5	f	29.1	2.0	1.0	1.0	0.0	0.0
B6-mtFVB	7 d	6	f	41.0	1.5	0.0	0.5	0.0	1.0

12-months-old mice

Mouse strain	Time of cerulein treatment	No.	Sex	Weight	Score AIP	Amylase (U/mL)	MPO (mU/mg lung)	ApopTag (cells/mm²)	CD11b (cells/mm²)
					Fig. 6, 7	Fig. 4	Fig. 5	Fig. 3	Fig. 3
B6-mtAKR	0 h	1	m	28.5	0	5.18	6.59	6.40	64.00
B6-mtAKR	0 h	2	m	34.6	0	2.78	8.70	0.00	44.80
B6-mtAKR	0 h	3	f	25.2	2	2.66	7.52	6.40	19.20
B6-mtAKR	0 h	4	m	38.4	2	3.61	8.98	6.40	51.20
B6-mtAKR	0 h	5	f	30.1	0	2.28	7.55	6.40	76.80
B6-mtAKR	0 h	6	f	26.4	0	2.27	10.42	12.80	96.00
B6-mtAKR	3 h	1	m	36.2	0	6.84			
B6-mtAKR	3 h	2	m	37.7	2	8.95			
B6-mtAKR	3 h	3	m	37.1	0	8.31			
B6-mtAKR	3 h	4	m	35.0	0	5.98			
B6-mtAKR	3 h	5	f	37.2	0	5.74			
B6-mtAKR	3 h	6	f	30.1	1	4.73			
B6-mtAKR	8 h	1	m	44.0	1	15.00	24.08	224.00	
B6-mtAKR	8 h	2	m	39.9	0	15.70	14.28	57.60	
B6-mtAKR	8 h	3	f	26.5	3	6.14	15.81	19.20	
B6-mtAKR	8 h	4	f	30.4	2	9.21	24.27	268.80	
B6-mtAKR	8 h	5	m	31.2	0	10.25	22.90	326.40	
B6-mtAKR	8 h	6	f	35.0	1	5.57	16.84	364.80	
B6-mtAKR	24 h	1	f	35.4	0	3.21	6.54	19.20	768.00
B6-mtAKR	24 h	2	m	42.4	1	4.82	8.53	19.20	1273.60
B6-mtAKR	24 h	3	f	25.4	0	4.22	4.63	25.60	1062.40
B6-mtAKR	24 h	4	f	29.6	1	4.04	10.19	19.20	3129.60
B6-mtAKR	24 h	5	m	34.4	2	5.29	9.10	38.40	2201.60
B6-mtAKR	24 h	6	m	36.1	2	4.66	16.06	25.60	1811.20
B6-mtAKR	7 d	1	m	39.0	2	3.09			
B6-mtAKR	7 d	2	m	40.2	0	3.44			
B6-mtAKR	7 d	3	m	39.4	1	3.08			
B6-mtAKR	7 d	4	f	24.5	0	2.21			
B6-mtAKR	7 d	5	f	23.8	2	2.25			
B6-mtAKR	7 d	6	f	24.8	2	2.43			

12-months-old mice

Mouse strain	Time of cerulein treatment	No.	Sex	Weight	Score AIP	Amylase (U/mL)	MPO (mU/mg lung)	ApopTag (cells/mm²)	CD11b (cells/mm²)
					Fig. 6, 7	Fig. 4	Fig. 5	Fig. 3	Fig. 3
B6-mtFVB	0 h	1	f	26.0	2	3.16	5.49	6.40	128.00
B6-mtFVB	0 h	2	m	38.2	3	4.08	6.45	0.00	57.60
B6-mtFVB	0 h	3	f	26.8	0	2.39	8.67	6.40	44.80
B6-mtFVB	0 h	4	m	35.5	0	2.79	10.04	6.40	25.60
B6-mtFVB	0 h	5	f	32.3	2	2.86	10.10	0.00	19.20
B6-mtFVB	0 h	6	m	41.5	0	3.14	12.18	0.00	25.60
B6-mtFVB	3 h	1	f	31.9	2	9.46			
B6-mtFVB	3 h	2	m	35.0	1	10.13			
B6-mtFVB	3 h	3	f	35.5	0	4.62			
B6-mtFVB	3 h	4	m	38.3	0	8.35			
B6-mtFVB	3 h	5	f	28.9	2	6.37			
B6-mtFVB	3 h	6	m	42.1	2	7.73			
B6-mtFVB	8 h	1	f	34.2	3	7.71	8.04	480.00	
B6-mtFVB	8 h	2	f	33.9	2	9.00	19.30	192.00	
B6-mtFVB	8 h	3	f	36.4	0	10.15	13.44	115.20	
B6-mtFVB	8 h	4	f	38.3	0	16.48	18.47	89.60	
B6-mtFVB	8 h	5	f	42.1	2	17.08	29.65	160.00	
B6-mtFVB	8 h	6	m	40.8	1	13.49	21.65	345.60	
B6-mtFVB	24 h	1	f	27.5	2	3.38	5.88	12.80	1152.00
B6-mtFVB	24 h	2	f	28.3	1	8.18	24.24	12.80	1158.40
B6-mtFVB	24 h	3	m	37.2	1	3.56	9.44	12.80	2521.60
B6-mtFVB	24 h	4	m	38.4	2	3.82	10.48	12.80	1676.80
B6-mtFVB	24 h	5	f	24.7	0	4.25	6.68	19.20	947.20
B6-mtFVB	24 h	6	m	42.7	1	4.23	17.91	6.40	787.20
B6-mtFVB	7 d	1	m	36.2	2	3.03			
B6-mtFVB	7 d	2	m	41.5	2	3.25			
B6-mtFVB	7 d	3	f	32.7	2	2.63			
B6-mtFVB	7 d	4	f	42.9	2	2.61			
B6-mtFVB	7 d	5	f	29.1	3	2.24			
B6-mtFVB	7 d	6	f	41.0	3	2.32			

24-months-old mice

Mouse strain	No.	Score AIP (Fig. 6)
B6-mtAKR	1	2
B6-mtAKR	2	2
B6-mtAKR	3	1
B6-mtAKR	4	2
B6-mtAKR	5	2
B6-mtAKR	6	2
B6-mtAKR	7	0
B6-mtAKR	8	2
B6-mtAKR	9	3
B6-mtAKR	10	3
B6-mtAKR	11	2
B6-mtAKR	12	1
B6-mtFVB	1	2
B6-mtFVB	2	2
B6-mtFVB	3	1
B6-mtFVB	4	2
B6-mtFVB	5	3
B6-mtFVB	6	2

12-months-old mice (acinar cells)

Pair of mice	Trypsin (Fig. 9)								Elastase (Fig. 9)								
	B6-mtAKR				B6-mtFVB				B6-mtAKR				B6-mtFVB				Cerulein treatment (time)
	0 min	30 min	60 min	90 min	0 min	30 min	60 min	90 min	0 min	30 min	60 min	90 min	0 min	30 min	60 min	90 min	
1	-0.35	1.81	6.56	3.71	-0.15	1.14	2.21	2.47	-0.14	3.78	7.75	12.51	0.78	3.05	8.19	13.08	ΔF/Δt
2	-0.40	5.62	8.74	8.19	0.09	5.40	6.90	14.63	-0.17	5.82	8.10	2.80	-0.08	6.22	7.59	4.30	
3	0.02	4.54	6.49	6.24	0.46	4.81	9.92	8.18	-0.25	5.08	7.73	10.52	-0.01	4.03	6.92	10.53	
4	0.09	5.37	9.30	9.46	-0.06	7.44	18.23	10.97	0.38	3.56	5.15	6.88	-0.06	7.44	18.23	10.97	
5	-0.25	2.33	11.08	1.43	0.63	3.99	16.47	13.37	-0.23	2.67	10.16	28.79	0.95	1.58	15.12	17.07	
6	1.07	1.78	19.77	11.83	0.44	1.89	5.98	8.01	-0.10	2.35	11.78	6.71	0.09	1.27	3.86	4.20	

Pair of mice	ROS (Fig. 10)						
	B6-mtAKR			B6-mtFVB			
	Cerulein			Cerulein			
	0	0.1 nM	10 nM	0	0.1 nM	10 nM	
1	2255	4710	1571	2684	5812	4311	Relative fluorescence units
2	1439	2375	274	3392	6777	754	
3	282	1029	823	767	492	1208	
4	553	354	412	1237	1361	1256	
5	2110	2143	1949	2034	1988	1944	
6	834	1069	1101	824	1347	974	
